# Tamoxifen-inducible cardiac-specific Cre transgenic mouse using *VIPR2* intron

**DOI:** 10.1186/s42826-020-00065-x

**Published:** 2020-09-15

**Authors:** Hyun Jung Chin, So-young Lee, Daekee Lee

**Affiliations:** Department of Life Science, Ewha Womans University, Ewhayeodae-gil 52, Seodaemun-gu, Seoul, 03760 South Korea

**Keywords:** VIPR2 intron, ERT2CreERT2, Heart, Tamoxifen-inducible Cre transgenic mouse

## Abstract

Genetically engineered mouse models through gene deletion are useful tools for analyzing gene function. To delete a gene in a certain tissue temporally, tissue-specific and tamoxifen-inducible Cre transgenic mice are generally used. Here, we generated transgenic mouse with cardiac-specific expression of Cre recombinase fused to a mutant estrogen ligand-binding domain (ERT2) on both N-terminal and C-terminal under the regulatory region of human vasoactive intestinal peptide receptor 2 (*VIPR2*) intron and *Hsp68* promoter (*VIPR2-ERT2CreERT2*). In *VIPR2-ERT2CreERT2* transgenic mice, mRNA for *Cre* gene was highly expressed in the heart. To further reveal heart-specific *Cre* expression, *VIPR2-ERT2CreERT2* mice mated with *ROSA26-lacZ* reporter mice were examined by X-gal staining. Results of X-gal staining revealed that Cre-dependent recombination occurred only in the heart after treatment with tamoxifen. Taken together, these results demonstrate that *VIPR2-ERT2CreERT2* transgenic mouse is a useful model to unveil a specific gene function in the heart.

## Introduction

The heart is the first organ to develop. It is also the most important organ for survival during embryonic development [1]. Gene knockout experiments have shown that abnormal development of the heart is the main cause of embryonic lethality [2], making it difficult to study specific gene function in the adult heart. To resolve this limitation, Cre/*loxP* recombination system has been developed to examine gene functions through gene deletion using adult mice [3, 4]. Such Cre/*loxP*-mediated conditional gene targeting can be used to study tissue-specific gene function, including genes expressed in the heart [5]. To activate Cre in a spatiotemporal manner, tamoxifen-inducible Cre/*loxP* recombination system requires Cre recombinase fused with a mutant form of estrogen receptor (CreERT2) which can migrate to the nucleus and induce site-specific recombination after treatment with tamoxifen [6]. Leaky Cre recombinase activity irrelevant to tamoxifen treatment can be more tightly controlled by placing the mutant estrogen receptor to both ends of Cre protein such as MerCreMer [7] and ERT2CreERT2 [8].

Regulatory DNA sequences are essential to achieve specific *Cre* expression. However, most *Cre* mouse lines express *Cre* in more than one specific tissue, leading to misinterpretation of data due to indirect effect of Cre-mediated gene deletion in other tissues on overall phenotype. In the present study, we selected an enhancer for heart-specific gene expression using VISTA enhancer browser and experimentally tested enhancer activity in transgenic mice [9]. Among these enhancers, hs1753 located in intron 4 of human *VIPR2* gene exhibited LacZ expression only in the heart, not in other region of embryonic day 11.5 (E11.5) mouse embryos. We generated a tamoxifen-inducible Cre transgenic mice using *VIPR2-ERT2CreERT2* expression vector consisting of hs1753 regulatory sequence, *Hsp68* promoter, *ERT2CreERT2* cDNA, and the polyadenylation site from the SV40 early region. We investigated whether Cre-dependent recombination occurs only in the heart of the embryo or adult after tamoxifen treatment on mice born by further crossing the *VIPR2-ERT2CreERT2* transgenic mouse with the *ROSA26-lacZ* reporter mouse.

## Materials and methods

### Construction of VIPR2-ERT2CreERT2 expression vector

Plasmid *pCAG-ERT2CreERT2* was a gift from Connie Cepko (Addgene plasmid # 13777). *pHsp68-LacZ-Gateway* was a gift from Nadav Ahituv (Addgene plasmid # 37843) and the plasmid *pCre-ERT2* was a gift from Pierre Chambon. All restriction enzymes and Phusion® high-fidelity DNA polymerase for PCR amplification were purchased from NEB (Ipswich, MA, USA). *Eco*R1-*Sac*I fragment (2.0 kb) and *Sac*I-*Sal*I (0.17 kb) fragment of *pCre-ERT2* were cloned to *Eco*R1 and *Sal*I sites of pBluescript II SK(+) to generate *pCre-ERT2pA* vector. A *Sma*I-*Cla*I fragment (1.8 kb) of *pCAG-ERT2CreERT2* was further cloned to *Sma*I and *Cla*I sites of *pCre-ERT2pA* to generate *pERT2CreERT2pA* vector without promoter or enhancer. Finally, *VIPR2*-driven *ERT2CreERT2* expression vector (*pVIPR2-ERT2CreERT2pA*) was constructed as follows. Human *VIPR2* genomic DNA located in intron 4 was amplified by PCR using a BAC clone (RP11-645 K21, BACPAC Resources, Oakland, CA, USA) with sense primer (5′-AAGCGGCCGCTGGGAGGAGAAGGGCTCTGC-3′, *Not*I site underlined) and antisense primer (5′-TTGACGCGTGAGAACAGGAGTGTCACCGG-3′; *Mlu*I site underlined). The PCR product (3.0 kb) was digested with *Not*I and *Mlu*I followed by purification with QIAquick PCR purification kit (Qiagen, Germany). The minimal promoter sequence of Hsp68 was amplified by PCR using a *pHsp68-LacZ-Gateway* (Ref: PubMed 17086198) with sense primer (5′- TTGACGCGTGAGCTTCCAGGAACATCCAAA-3′, *Mlu*I site underlined) and antisense primer (5′-AATCTAGACGCTCTGCTTCTGGAAGGCT-3′, *Xba*I site underlined). The PCR product (0.9 kb) was digested with *Mlu*I and *Xba*I followed by purification with QIAquick PCR purification kit. Both fragments were cloned to *Not*I and *Spe*I sites of *pERT2CreERT2pA* to obtain *pVIPR2-ERT2CreERT2pA*. 5′ VIPR2 sequence of *pVIPR2-ERT2CreERT2pA* was verified by DNA sequencing. *Not*I-*Kpn*I fragment of *VIPR2-ERT2CreERT2* expression vector was purified with QIAquick gel extraction kit (Qiagen) and dissolved in TE buffer (10 mM Tris-HCl, pH 7.4, 0.25 mM EDTA) to 1 μg/ml for microinjection.

### Generation and maintenance of transgenic mice

Three-week-old female FVB/N mice were injected intraperitoneally with pregnant mare serum gonadotropin (Merck KGaA, Germany) and human chorionic gonadotropin (hCG, Merck KGaA) 48 h later. After administration of hCG, female mice were mated with FVB/N male mice. The ampulla region of oviduct from plugged females was teared with a fine needle in 1x PBS containing 0.1% hyaluronidase (Merck KGaA) and 0.1% polyvinylpyrrolidone (M.W. 40 kDa, Merck KGaA). Fertilized eggs were collected using mouth-controlled pipet. These eggs were washed several times with M2 medium (Merck KGaA) and cultured in the drop of KSOM medium (Merck KGaA) under mineral oil (Merck KGaA) until microinjection. After pronuclear injection of DNA, eggs survived microinjection were then transferred into oviducts of pseudopregnant ICR females. Founder mice containing injection gene were identified with PCR genotyping. Subsequent generation of transgenic mice were maintained congenic on C57BL/6 J genetic background. All mice experiments were approved by the Institutional Animal Care and Use Committee (IACUC) of Ewha Womans University (Permit number: 2015–01-072).

### PCR genotyping

Toe clips from 7 to 10 days old pups were used to extract DNA for PCR genotyping with 4 primers. PCR primers Hsp68-S1 (5′-CAGGTCACCAGACGCTGACA-3′) and ERT2-AS1 (5′-TCATGTCTCCAGCCATGGTG-3′) produced a 276-bp PCR product specific for transgene while PCR primers Ereg-S3 and Ereg-AS1 primers produced a 159-bp PCR product from *Ereg* gene for endogenous control [10]. PCR product was resolved with 1.2% agarose gel electrophoresis and visualized with ethidium bromide staining.

### RNA preparation, reverse-transcription (RT), and quantitative RT-PCR (qRT-PCR)

After euthanasia of mouse with CO_2_, tissues were isolated and immediately frozen in liquid nitrogen. Total RNA was prepared using TRIzol Reagent (Thermo Fisher Scientific, Waltham, MA, USA) following the manufacturer’s protocol. To remove any residual DNA in RNA preparation, RNA solution was treated with DNaseI (Thermo Fisher Scientific) at 37 °C for 30 min and followed by RNA clean up with RNeasy MinElute Cleanup Kit (Qiagen, Germany). Then cDNA was generated from RNA using Superscript III reverse transcriptase (Thermo Fisher Scientific) and random hexamers as described previously [10]. Reverse transcription products were amplified by real-time PCR with *Cre*-S (5′-GGCATGGTGCAAGTTGAAT-3′) and *Cre*-AS (5′-AGCATTGCTGTCACTTGGTC-3′); *Il6*-S (5′-ATGAGAAAAGAGTTGTGCAATGGC-3′) and *Il6*-AS (5′-CCAGGTAGCTATGGTACTCCAGAA-3′); *Col3a1*-S (5′-GATGAGCTTTGTGCAAAGTGG-3′) and *Col3a1*-AS (5′-CGCAAAGGACAGATCCTGA-3′) primers. *18S rRNA*-S (5′-TCAACTTTCGATGGTAGTCGCC-3′) and *18S rRNA*-AS (5′-GGCCTCGAAAGAGTCCTGTATTGT-3′) primers were used to determine expression level of *18S rRNA* as endogenous control. Real-time PCR was performed with KAPA SYBR FAST ABI Prism qPCR Kit (KAPA Biosystems, Wilmington, MA, USA) using ABI Prism 7300 (Thermo Fisher Scientific) machine. The relative amount of transcript was quantified as described previously [11].

### Tamoxifen treatment

Tamoxifen (Merck KGaA) was dissolved in ethanol to 100 mg/ml or 200 mg/ml and further diluted with sunflower seed oil (Merck KGaA) to 10 mg/ml and 20 mg/ml, respectively [12]. For collection of embryos, pregnant mice at 9 days post coitum were injected intraperitoneally with 0.1 ml of 10 mg/ml tamoxifen solution for three consecutive days. Embryos were harvested at 1 day or 3 days after the last injection. For collection of adult tissues, mice (3 to 5 months old) were injected intraperitoneally with 0.1 ml of 20 mg/ml tamoxifen for five consecutive days and tissues were harvested at 7 days after the final injection. Ethanol in sunflower seed oil at identical concentration was injected intraperitoneally as a negative control.

### LacZ staining

Embryos were collected from pregnant *Gtrosa26*^*tm1Sor/tm1Sor*^ females mated with *VIPR2-ERT2CreERT2* males and stained with X-gal as described previously [13]. Whole-mount embryos were imaged using a SMZ 1000 dissecting microscope (Nikon). Tissues from adult mice derived from breeding *Gtrosa26*^*tm1Sor/tm1Sor*^ females with *VIPR2-ERT2CreERT2*^*Tg/+*^ males were collected, fixed, and stained with X-gal as described previously [10]. Slides were analyzed using a Nikon Eclipse 80i microscope and images were captured with a DS-Ri1 camera (Nikon).

### Statistical analysis

Experimental groups were compared with one-way ANOVA with Turkey’s multiple comparison test using GraphPad Prism program (GraphPad Software, San Diego, CA, USA).

## Results and discussion

### Tamoxifen-dependent Cre activity in embryonic heart of transgenic mice

**Fig. 1:**
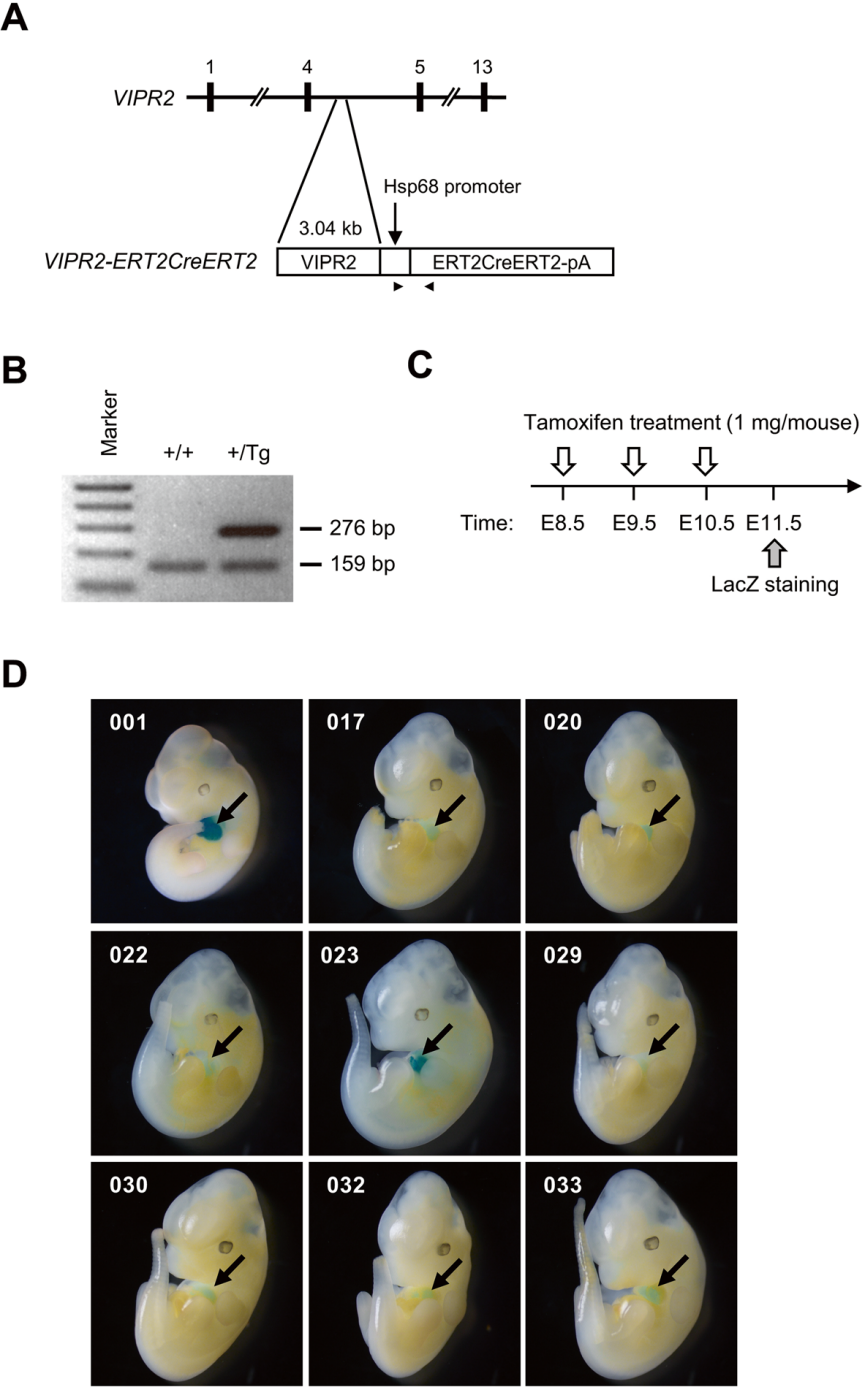
Generation of *VIPR2-ERT2CreERT2* transgenic mice and analysis of transgene expression. **a** Schematic structure of *VIPR2-ERT2CreERT2* transgene. Numbers indicate exons of human *VIPR2* gene. Arrowheads indicate primers for genotyping. **b** PCR genotyping of representative founder mice showing a 276-bp PCR product specific for transgene and a 159-bp product for endogenous gene (+/+, wild-type; +/Tg, heterozygous transgenic mouse). **c** Strategies for treating pregnant mice with tamoxifen followed by LacZ staining. **d** Whole-mount LacZ staining of *VIPR2:R26* compound heterozygotes E11.5 embryos. Each number indicates *VIPR2-ERT2CreERT2* transgenic mouse line. Arrows indicate heart-specific X-gal staining. Original magnification, 20 ×

**Fig. 2:**
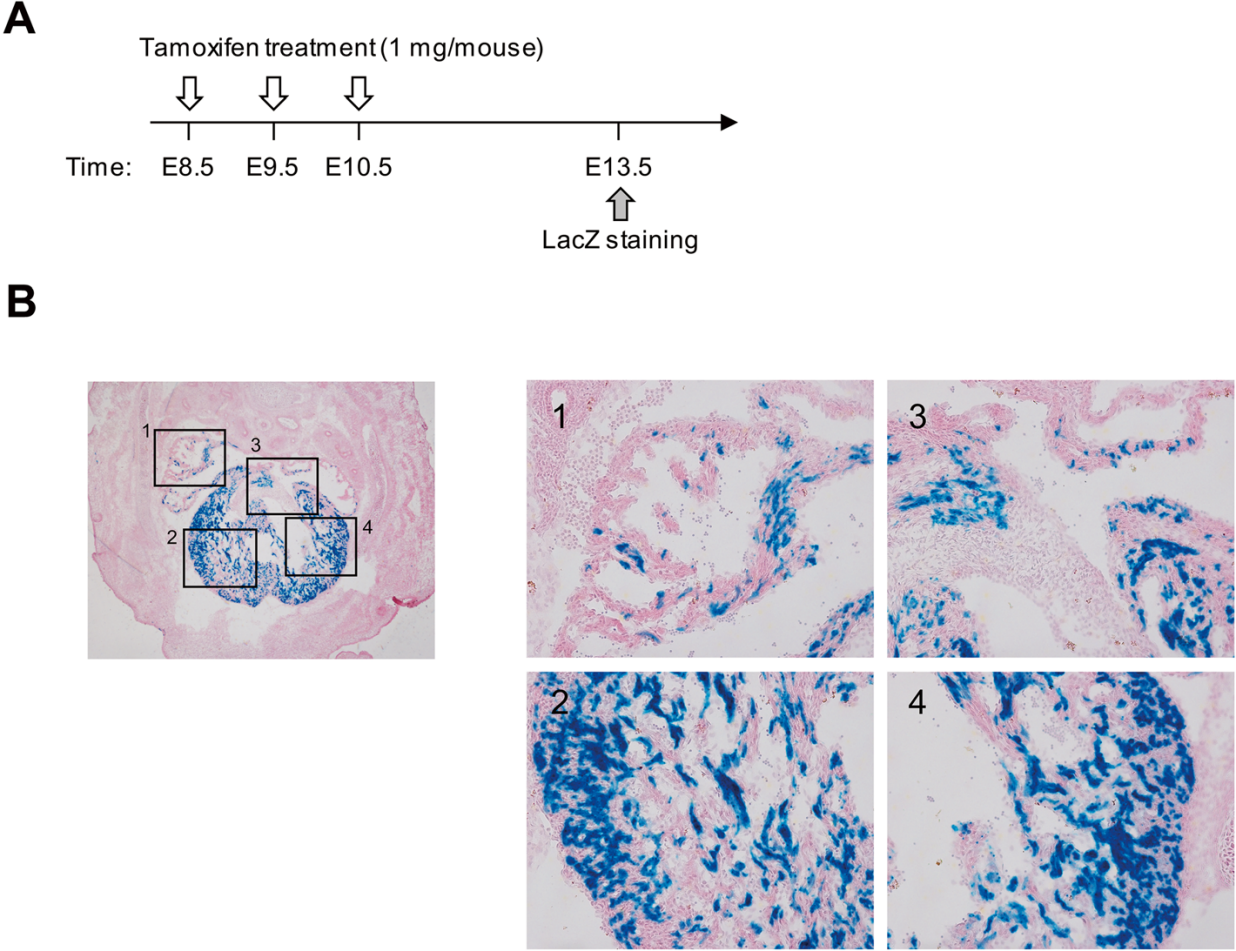
LacZ staining of E13.5 embryos. **a** Strategies for treating pregnant mice (*Gtrosa26*^*tm1Sor/tm1Sor*^ mated with *VIPR2-ERT2CreERT2*^*Tg/+*^) with tamoxifen followed by LacZ staining. **b** Frozen section was stained with X-gal followed by counterstaining with hematoxylin and eosin Y (H&E) briefly. Left, original magnification, 40×; Right, 100× magnification of rectangle 1 to 4. Scale bar, 50 μm

VISTA enhancer browser was used to identify enhancer for heart-specific gene expression [9]. Based on LacZ staining of E11.5 embryos (https://enhancer.lbl.gov), hs1753 (chr7:158,888,320-158,891,362) located in intron 4 of human *VIPR2* gene was subcloned into *Hsp68* promoter fused with *ERT2CreERT2-pA* to generate *VIPR2-ERT2CreERT2* expression vector (Fig. 1a). A total of nine transgenic founders were obtained out of 22 newborn pups. Subsequent generations of transgenic mice were identified by PCR genotyping (Fig. 1b). To determine *Cre* expression, transgenic males mated with homozygous *Gtrosa26*^*tm1Sor*^ [14] females were examined for the expression of LacZ due to excision of *Gtrosa26*^*tm1Sor*^ by tamoxifen-dependent Cre recombinase. After three consecutive injections of tamoxifen, whole embryos were stained with X-gal (Fig. 1c). All transgenic lines showed heart-specific LacZ staining only in *VIPR2-ERT2CreERT2*^*Tg/+*^ and *Gtrosa26*^*tm1Sor/+*^ compound heterozygous (*VIPR2:R26*) embryos (Fig. 1d). Because VIPR2-ERT2CreERT2–001 line exhibited the strongest expression of LacZ, further studies were performed with this line as a representative. To confirm the specificity of Cre recombination in the heart, we analyzed *VIPR2:R26* E13.5 embryo sections after treating pregnant mice with tamoxifen (Fig. 2a). Heart-specific LacZ expression was detected in the whole region of the heart with punctate pattern (Fig. 2b). These results indicate that the 3.04-kb of *VIPR2* intron 4 harbors cis-regulatory elements for heart-specific expression during embryonic development.

### Heart-specific Cre activity in adult VIPR2-ERT2CreERT2 transgenic mice

**Fig. 3:**
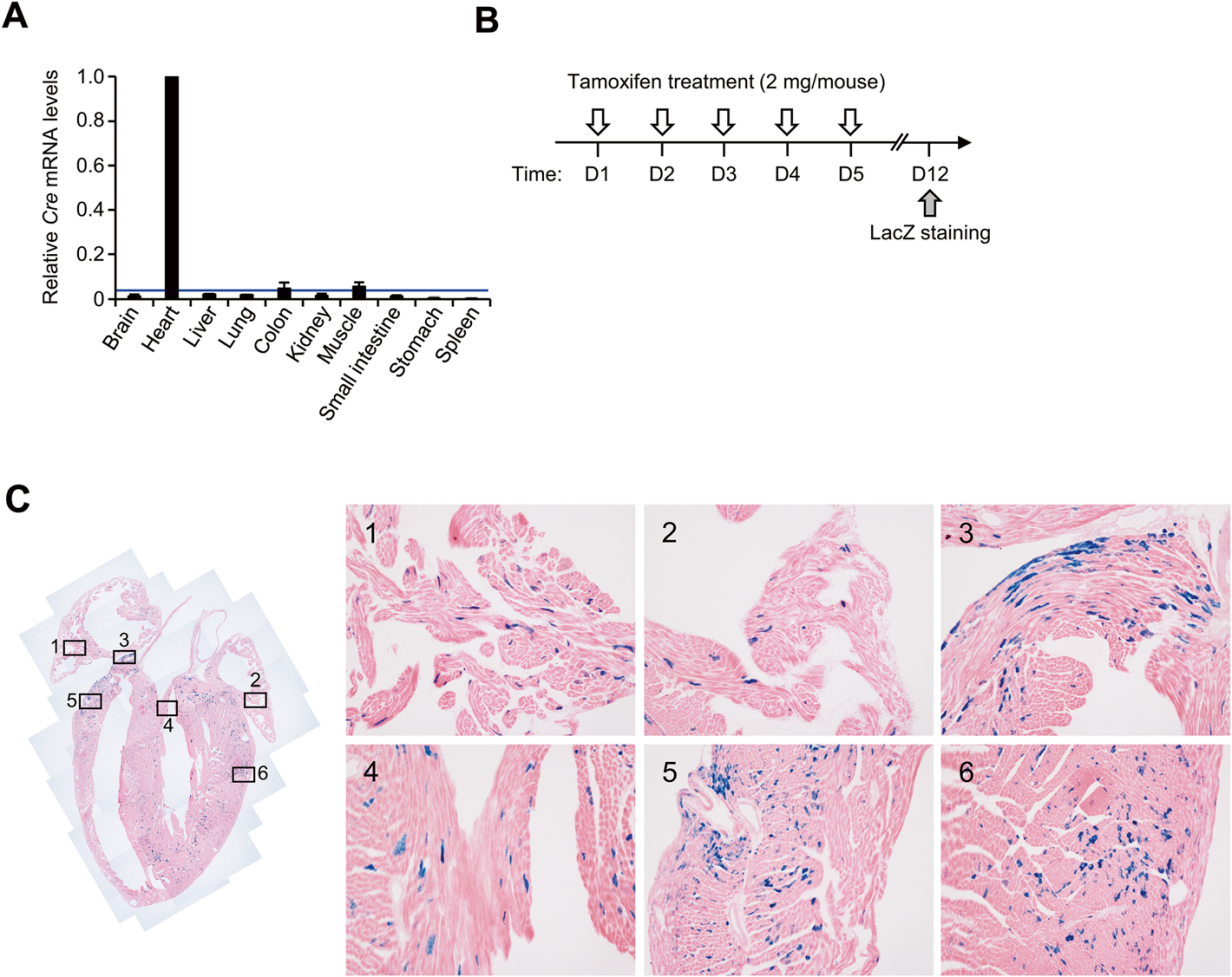
Analysis of tissue-specific Cre activity. **a** qRT-PCR analysis of relative *Cre* mRNA levels in various tissues. Total RNA prepared from *VIPR2-ERT2CreERT2*^*Tg/+*^ adult mice was analyzed by real-time PCR and the relative *Cre* mRNA level in each tissue was compared to the level of the heart (*n* = 2). The blue line indicates the non-specific *Cre* mRNA level of’ the wild-type heart. Data are presented as mean ± SD. Only the level of the heart is statistically significant (*p* < 0.01). **b** Strategies for treating adult *VIPR2:R26* compound heterozygotes with tamoxifen followed by LacZ staining. **c** Frozen sections of the heart were stained with X-gal followed by counterstaining with H&E briefly. 1, right atrium; 2 and 3, left atrium; 4, aortic valve; 5, right ventricle; 6, left ventricle. Original magnification, 40×; Right, 100× magnification of rectangles 1 to 6

**Fig. 4:**
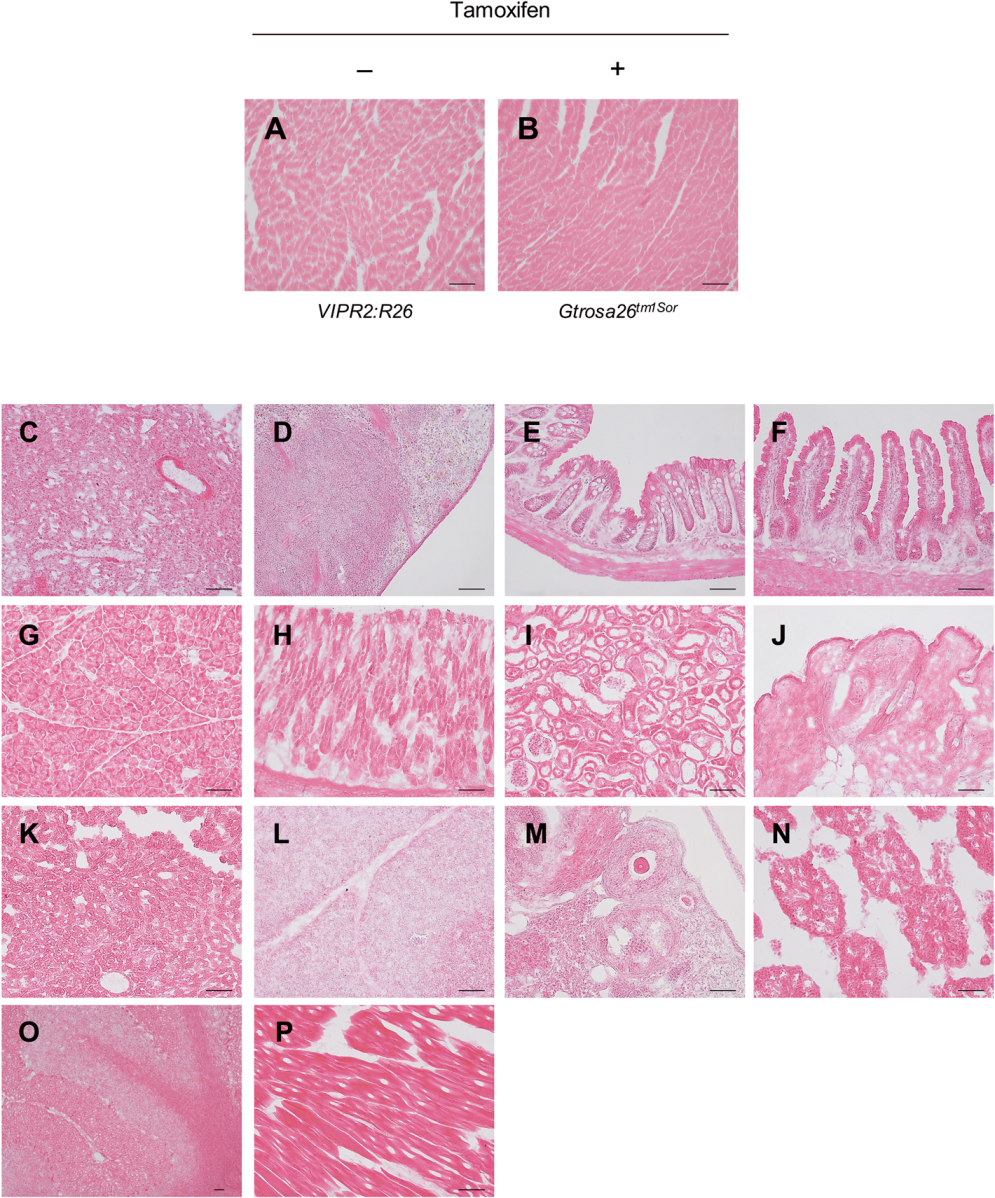
Analysis of tissue-specific Cre activity by LacZ staining. Frozen sections were stained with X-gal followed by counterstaining with H&E briefly. **a** Heart section of adult *VIPR2:R26* compound heterozygote mouse without tamoxifen treatment. **b** Heart section of adult *Gtrosa26*^*tm1Sor/+*^ treated with tamoxifen; **c-p** Adult *VIPR2:R26* compound heterozygote mouse treated with tamoxifen: **c** Lung; **d** Spleen; **e** Colon; **f** Small intestine; **g** Pancreas; **h** Stomach; **i** Kidney; **j** Skin; **k** Liver; **l** Thymus; **m** Ovary; **n** Testis; **o** Brain; **p** Muscle. Scale bar, 50 μm

**Fig. 5:**
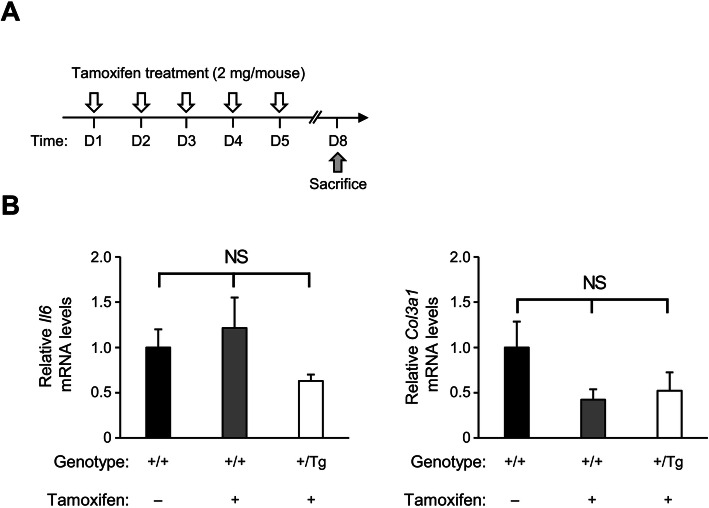
Analysis of short-term treatment of tamoxifen on heart. **a** Strategies for treating adult *VIPR2-ERT2CreERT2*^*Tg/+*^ and wild-type mice with tamoxifen. **b** and **c** Total RNA prepared from each group of mice (*n* = 4) was analyzed by qRT-PCR. Relative *Il-6* mRNA and *Col3a1* mRNA levels in the heart were then compared. Data are presented as mean ± SEM. NS, statistically insignificant, one-way ANOVA

Before analyzing LacZ activity in adult *VIPR2:R26* mouse, we determined *Cre* mRNA levels in various organs of *VIPR2-ERT2CreERT2–*001 line. Relative expression levels of *Cre* mRNA were basal in most organs compared to those in the heart (Fig. 3a). To further confirm tamoxifen induced Cre recombination, *VIPR2:R26* adult mice were treated with either 2 mg of tamoxifen or the same volume of ethanol with sunflower oil mixture (1:9, v/v) as control injection for five consecutive days. Organs were harvested 7 days after the last injection (Fig. 3b). LacZ staining was detected mainly in cardiomyocytes of all regions with punctate pattern similar to that in the embryo (Fig. 3c). In contrast, LacZ staining was not observed in the heart of *VIPR2:R26* adult mouse treated with solvent alone or *Gtrosa26*^*tm1Sor/+*^ mouse treated with tamoxifen solution (Fig. 4a, b). The heart was the only organ stained with X-gal while all other organs were negative for the X-gal staining (Fig. 4c to p). The degree of X-gal staining of the heart did not differ by gender. Moreover, consecutive treatment of *VIPR2-ERT2CreERT2* transgenic mouse with tamoxifen did not show harmful effects such as inflammatory response or heart reorganization (Fig. 5a to c). Overall, these results indicate that *VIPR2-ERT2CreERT2* transgenic mouse is useful for heart-specific gene knockout experiment using adult mice without leaky expression or adverse effect.

The adult heart is composed of several cell types, including cardiomyocytes, cardiac fibroblasts, vascular smooth muscle cells, and endothelial cells. Fluorescence-activated cell sorting (FACS) analysis has revealed that cardiomyocytes, the majority of cell types, account for 56% of heart cell types while the rest of heart cells are cardiac fibroblasts (27%), vascular smooth muscle cells (10%), and endothelial cells (7%) [15]. In the present study, cardiomyocytes were the major cell types for expressing LacZ while vascular smooth muscle cells and endothelial cells were rare in the heart of transgenic mice. The most widely used gene for driving heart-specific *Cre* expression is α-myosin heavy chain (*Myh6*) [16]. Inducible Cre recombinase using *Myh6* promoter has also been established [17, 18]. However, adverse effect of tamoxifen treatment on normal heart function in *Myh6-MerCreMer* mice requires appropriate control mice to avoid misinterpretation of conditional knockout mice phenotype [19, 20]. Recently developed *Cre* knock-in mouse by targeting a MerCreMer cassette into the start codon of *Myh6* seems to be able to replace *Myh6-MerCreMer* mice since these knock-in mice exhibit normal heart function regardless of tamoxifen treatment [21]. In contrast to robust recombination in cardiomyocytes of *Myh6* promoter-driven *Cre* lines, *VIPR2-ERT2CreERT2* transgenic mouse exhibited far less recombination in the heart, suggesting that this transgenic mouse could be useful for analyzing genes whose cardiomyocytes-wide deletion might lead to heart failure.

## Conclusions

LacZ staining after crossing with *Rosa26-lacZ* reporter mouse revealed that *VIPR2-ERT2CreERT2* transgenic mice showed tamoxifen-dependent Cre activity only in the heart. This transgenic mouse can be used as a tool to temporally and spatially target genes in the heart and then, making it a useful model for revealing specific gene functions in the heart.

## Data Availability

*VIPR2-ERT2CreERT2–*001 transgenic mouse was backcrossed with C57BL/6 J mice for 10 generations, and then deposited in the Korea National Institute of Food and Drug Safety Evaluation for distribution [Stock number, 18-NIFDS-M-ET-001; Stock name, B6.Cg-Tg (VIPR2-ERT2CreERT2)^Dkl^/Korl].

## Abbreviations

CreERT2: Cre recombinase fused with a mutant form of estrogen receptor
E11.5: Embryonic day 11.5
ERT2: Mutant form of estrogen receptor
FACS: Fluorescence-activated cell sorting
H&E: Hematoxylin and eosin Y
hCG: Human chorionic gonadotropin.
IACUC: Institutional Animal Care and Use Committee
Myh6: α-myosin heavy chain
qRT-PCR: Quantitative RT-PCR
RT: Reverse-transcription
VIPR2: Vasoactive intestinal peptide receptor 2
